# National emergency X-radiography utilization study guidelines versus Canadian C-Spine guidelines on trauma patients, a prospective analytical study

**DOI:** 10.1371/journal.pone.0206283

**Published:** 2018-11-02

**Authors:** Alireza Ala, Samad Shams Vahdati, Amir Ghaffarzad, Haleh Mousavi, Mohammad Mirza-Aghazadeh-Attari

**Affiliations:** 1 Emergency Department, Emergency Medicine Research Team, Tabriz University of Medical Science, Tabriz, Iran; 2 Road Traffic Injury Research Center, Tabriz University of Medical Science, Tabriz, Iran; 3 Emergency medicine research team, Tabriz University of Medical Science, Tabriz, Iran; 4 Emergency Department, Emergency Medicine Research Team, Tabriz University of Medical Science, Tabriz, Iran; 5 Student research committee, Tabriz University of Medical Sciences, Tabriz, Iran; University of Utah Hospital, UNITED STATES

## Abstract

**Introduction:**

The most common cause of hospital emergency department visits is trauma resulting from a variety of underlying mechanisms. Unknown neck and spinal cord injuries and a lack of early diagnosis can have catastrophic consequences, such as paralysis of some or all limbs. The use of imaging techniques reduces the number of patients suffering from severe injuries.

**Objective:**

To assess and compare the effectiveness and ease of utilizing two different sets of guidelines, the National Emergency X-Radiography Utilization Study guidelines (NEXUS) and the Canadian C-Spine guidelines (CCR), on trauma patients.

**Methods:**

This study was approved by the Ethics Committee of Tabriz University of Medical Sciences. Of all the patients presenting to the hospital, 200 trauma patients were randomly included in the study. NEXUS and CCR were surveyed for each patient, and subsequent radiographies were also requested. The specificity and sensitivity of each of the methods was calculated, and the two methods were compared using Kendall’s W test.

**Results:**

A total of 200 trauma patients who met the inclusion criteria were included in the study. A total of 69.5% of the patients were male, and 30.5% were female. According to NEXUS guidelines, 47.5% of the patients were required to undergo neck radiography. According to CCR guidelines, 57.5% of the patients were required to undergo neck radiography. The sensitivity was found to be 90% for neck radiography by both NEXUS and CCR guidelines, while specificities were found to be 54.73% and 44.2% for NEXUS and CCR guidelines, respectively.

**Conclusion:**

This study showed that the two guidelines have the same sensitivity for evaluating which trauma patients need to undergo radiography. It seems that the NEXUS guidelines have the same effectiveness as CCR for determining which trauma patients need to undergo radiography. They also perform better than CCR guidelines in terms of ruling out which cases need no further radiologic investigation.

## Introduction

Trauma cases are definitively the most common cause of hospital emergency department visits. Blunt trauma is among the most dangerous types of trauma due to the likelihood of damage in various organs, such as the neck and spinal cord [1, 2]. According to statistical analysis, 13 million trauma patients are treated annually with the possibility of cervical spinal cord injury in the United States and Canada [3, 4].

Cervical spine injuries occur frequently in cases of major trauma. In developed countries, the incidence of traumatic spinal injury is decreasing, but the same is not true for developing countries, in which the total numbers remain high and are largely caused by traffic accidents and falling from heights [5]. Detecting cervical spine stability at the time of injury with the reduced consciousness that typically accompanies major trauma incidents is challenging [6]. Hence, determining the type of radiography and selection criteria for radiography is important.

Unrecognized neck and spinal cord injuries resulting from trauma and lack of early diagnosis can have catastrophic consequences, such as paralysis of some or all limbs [7]. This causes emergency room physicians, especially emergency specialists, to request neck radiology as the first diagnostic step for trauma patients. Most of these requested radiographies are normal, and only a handful show signs of clinically significant abnormality. The previously mentioned issues raise concerns regarding the effectiveness of the common approach in detecting cervical injuries, and lead to problems such as misdiagnosis, over diagnosis and unnecessarily increased financial burden [8–12]. Although radiographies are a relatively simple and inexpensive method for detecting cervical injuries compared to other techniques, their abundant use imposes an enormous burden on the health system [13, 14]. In addition to the financial costs, immobility of the patients when waiting for the X-ray not only causes discomfort for patients and their companions but also leads to an unnecessary accumulation and occupation of beds in the emergency room [15–17]. All of these problems have caused numerous and significant conflicts and differences of opinion between clinicians, which has led to the publishing of contradicting guidelines on the use of simple radiographies. For convenient and efficient clinical decisions, especially in cases similar to this one, we can use a variety of related studies to design a chart to help physicians make a decision based on the variables of the examination and simple tests [18–22].

NEXUS guidelines were first introduced in 1992 and included the following five criteria [23]:

1. Absence of tenderness when touching the cervical spine
2. Absence of evidence of intoxication
3. Full consciousness
4. Absence of focal neurological lesions
5. Absence of damage causing distraction

According to these guidelines, in cases in which all of the above are present, there is no need to take lateral neck radiographs in trauma patients [24]. According to a study by Hoffman et al., National Emergency X-Radiography Utilization Low-Risk Criteria (NLC) exhibits 99.6% sensitivity and 12.9% specificity in detecting fractures, which has caused doctors to recommend using this method [25]. In the past decade, a group of emergency physicians in the city of Ottawa, Canada, introduced the CCR (Canadian C-spine Rule) evaluation method. CCR guidelines have three criteria for the assessment of patients with neck trauma that specify whether a patient requires neck radiography [26]. Similar to the previous method, this method evaluates the patient's condition and his or her need for imaging according to 3 high-risk criteria, 5 low-risk criteria, and the patient's ability to rotate the head [27–29]. Goddard reported in a study that CCR is superior in reducing unnecessary radiographic imaging in conscious adults with stable vital signs and cervical spine injuries compared to typical clinical judgment [30]. Ian et al. showed that CCR has higher sensitivity and specificity and reduces the number of patients requiring radiography [31]. In a study conducted by Stiell et al., 2% of subjects had a neck injury. The study showed that in patients with complete consciousness, CCR was more sensitive in diagnosing fractures [32]. Zoe Michaleff et al. demonstrated that CCR guidelines are more sensitive than NEXUS guidelines [33]. Many studies have investigated the methods for evaluating trauma patients in order to determine under what circumstances X-rays should be performed. Therefore the choice that which guidelines better assist the assessment and can be easily implemented are always controversial [34, 35]. Therefore, we decided to assess the relative sensitivity and specificity of the two methods in a referral center in a developing country, in which access to biomedical imaging is not without challenges.

## Materials and methods

### Study design and setting

This is an analytic, descriptive, prospective, double-blinded study focusing on 2 major guidelines for assessing cervical trauma: NEXUS and CCR. Of the trauma patients admitted to the Emergency Department of Imam Reza Medical Educational Center (a trauma referral center for the Tabriz greater metropolitan area, and the referral center for areas northwest of Iran, equipped with CT scan, MRI, PET scan, and attending physicians from various specialties and subspecialties; it has an estimated number of 10000 trauma cases each year), 200 were randomly selected (by simple randomization) using Excel software and enrolled in the study. The selection was done in all hours of the day, and randomization was done via فاث table of random numbers.

### Inclusion and exclusion

Inclusion criteria were as follows: age >18, head and neck trauma, stable vital signs, GSC = 15 during the first 48 hours after the initial trauma. Exclusion criteria included age <18, penetrating neck trauma, acute paralysis, known disease or abnormality of the spine, pregnancy, visible damage to the clavicle, high impact trauma (either recorded by EMS personnel or self-reported by patients) and having been discharged by personal request prior to completion of the necessary medical procedures.

### Survey via protocols

Prior to initiation, during a one-hour session, medical staff were briefed on the study protocol (all personnel had previous experience in utilizing both guidelines). Considering that the guidelines consist of a check list, clinicians only needed to follow the check list, resulting in no possible disagreements on account of limited options. After the patients entered the emergency department, the initial evaluation was carried out by emergency attendants and emergency medicine residents. The clinical findings were registered in the patients' electronic medical records, and the presence or absence of obvious spinal injury was determined by both guidelines. Simple X-ray imaging was performed for all trauma patients to rule out neck injury. If prescribed, computerized tomography was performed to assess the damage to the spine. Radiographic images were interpreted by board certified radiologists. The radiologists were aware of the clinical status of the patients, but they were not aware of the study protocol. The emergency attending clinicians and residents did not have any information about the imaging results until the initial surveys were finished. A third party was responsible for following the patients’ treatment outcomes and was not informed about the patients’ prior diagnostic information. Finally, three cervical view X-rays were obtained (AP, lateral, and open mouth) for all patients, and in cases of high clinical divergence from radiological findings, further imaging was requested. Finally, the results obtained from both sets of guidelines were compared with each other.

### Statistical analysis

The results of the patients’ evaluation using both CCR and NEXUS-NLC guidelines were recorded in a check list. The accuracy of the two guidelines was examined using indicators of sensitivity and specificity. The gold standard is to compare the results of the two guidelines, the simple graphs report, and the patients’ CT scan. All data were analyzed using SPSS version 15 using descriptive analysis, Mean±SD and frequency tables. Guidelines were compared using Kendall’s W test.

### Ethical considerations

This study was approved by the Research Committee of Tabriz University of Medical Sciences and received the approval of the ethics committee of the University (ethics committee code: NO:5/4/7828 Date:2015/12/19).

## Results

In this study, 200 trauma patients admitted to the Emergency Department of Tabriz Imam Reza Hospital were randomly selected. Of the 200 patients included, 139 (69.5%) patients were male and 61 (30.5%) were female. Mean ± SD of age of patients was 40.00 ± 17.75 years (CI: 95% - 19,84). Mean ± SD age of women and men were 42.56 ± 20.8 years (CI: 95% - 19,84) and 38.88 ± 16.51 years (CI:95% - 19,84), respectively.

The frequencies obtained for the mechanism of trauma for each case are presented in Table 1.

**Table 1:** Frequencies of the trauma mechanism in the patients studied.

	Frequency	Percentage
Car accident	44	22
Pedestrian accident	34	17
Rollover	32	16
Fall from height	29	14.5
Motorcycle-car accident	24	12
Fall	21	10.5
Dispute	12	6
Staying under rubble	2	1

The most frequent trauma mechanisms were car accidents, pedestrian accidents, rollovers, and falls from heights.

The results for the survey conducted according to the NEXUS guidelines are presented in Table 2.

**Table 2:** Frequency of different items discussed in NEXUS guidelines.

	Frequency	Percentage
Cervical spine tenderness	35	17.5
Evidence of intoxication	2	1
Normal level of consciousness	162	81
Focal neurologic deficits	5	2.5
Painful misleading injury	70	35

As shown in Table 2, 162 (81%) patients had a normal level of consciousness, while only 5 patients (2.5%) had a focal neurological deficit. In addition, 35 (17.5%) patients had cervical vertebral tenderness, and 70 of them had concurrent painful misleading injury.

The results for the survey conducted by the CCR guidelines are shown in Table 3.

**Table 3:** Frequency of different items discussed in CCR guidelines.

	Frequency	Percentage
Age greater than or equal to 65 years	26	13
Severe damage mechanism	127	63.5
Paresthesias in extremities	5	2.5
Simple rear vehicle collision	24	12
Sitting position in emergency	58	29
Outpatient status at any time after trauma	43	21.5
Delayed onset of neck pain	14	7
Cervical spine tenderness	34	17
The ability to rotate neck 45 degrees to left and right	144	72

As shown in Table 3, 127 (73.5%) patients suffered severe damage on the basis of CCR guidelines, but only 24 (12%) of them mentioned a rear vehicle collision. Moreover, 34 patients (17%) had cervical spine tenderness, but 144 patients (72%) had the ability to rotate their necks 45 degrees to the right and left.

The results regarding whether to pursue radiography according to the NEXUS guidelines are shown in Table 4.

**Table 4:** Frequency of the results obtained for radiography based on NEXUS and CCR guidelines.

	Frequency	Percentage	P-value
NEXUS	95	47.5	0.004*
CCR	115	57.5	

*Kendall’s W test

As shown in Table 4, based on the NEXUS guidelines, 95 (47.5%) patients needed radiography, and according to CCR guidelines, 115 (57.5%) of them required radiography. A significant correlation was found between the two guidelines (p-value <0.001).

According to the results obtained from neck radiography, only 10 (5%) patients had neck injury in the cervical spine, and 190 (95%) patients had no cervical spine injuries.

The analysis of data for the true- and false-positive and true- and false-negative reviews using NEXUS and CCR guidelines are shown in Table 5.

**Table 5:** True- and false-positive and true- and false-negative reviews using NEXUS and CCR guidelines.

	Frequency (%)	
	NEXUS	CCR
True-positive	9 (4.5%)	9 (4.5%)
True-negative	104 (52%)	84 (42%)
False-positive	86 (43%)	106 (53%)
False-negative	1 (0.5%)	1 (0.5%)

**Fig 1:**
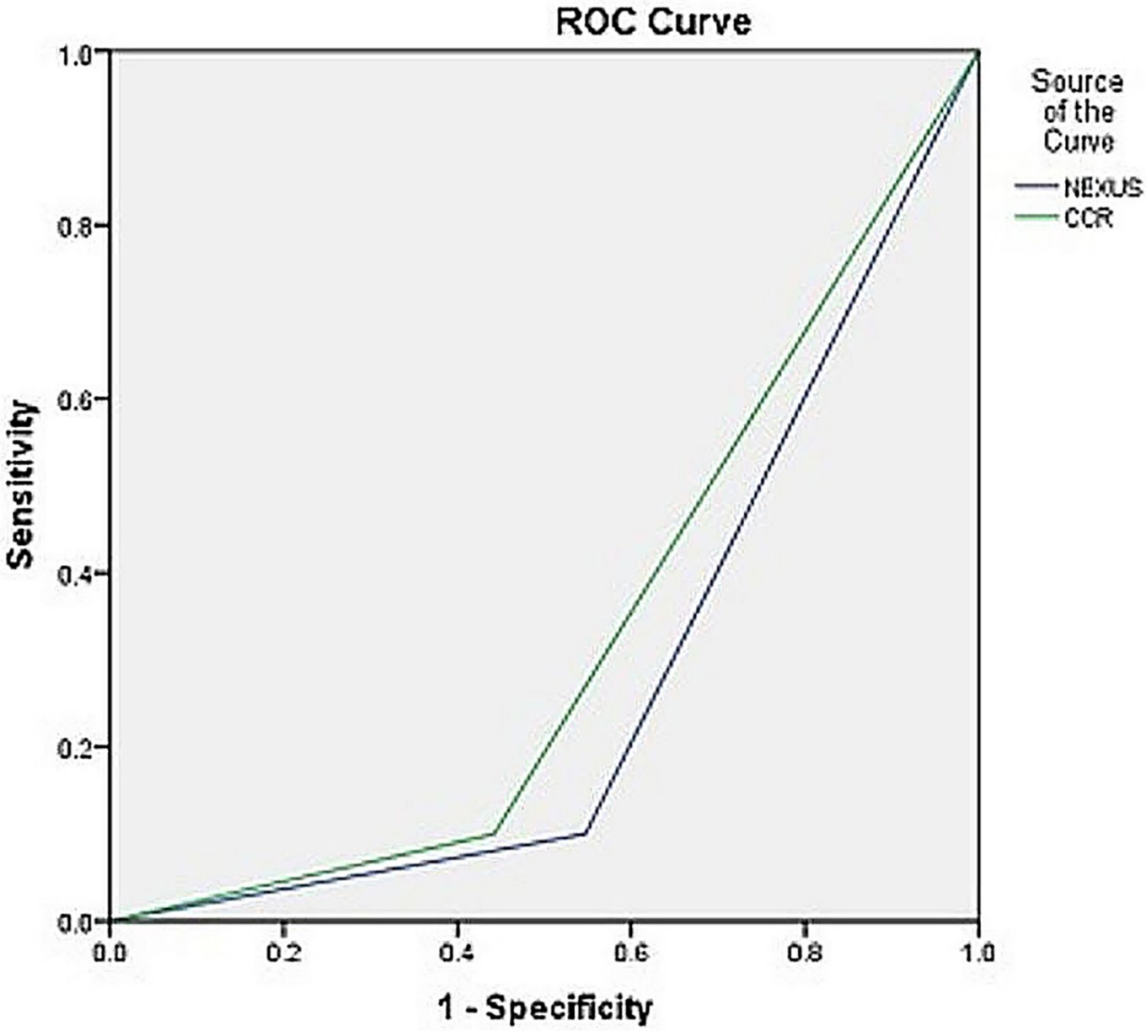
ROC curve for specificity and sensitivity.

As shown in Table 5, true-positive and false-negative rates are similar for both guidelines, and they are equal to 4.5% and 1%, respectively. In the survey conducted regarding sensitivity and specificity criteria for neck radiography by NEXUS and CCR guidelines, a sensitivity of 90% was obtained for both guidelines, while the specificity was found to be 54.73% and 44.2% for NEXUS and CCR guidelines, respectively (Fig 1).

## Discussion

In the present study, we examined 200 trauma patients who were referred to Imam Reza Hospital Emergency Department. All the patients underwent cervical spine radiography. Ten (0.5%) showed evidence of cervical spine injury. In a study conducted by Stiell et al. (2003), the prevalence of cervical injury was reported to be 2%. The difference in the prevalence of cervical injury in these two studies could be accounted for by differing trauma mechanisms. In this study, 200 patients were examined, but 8283 patients were evaluated in the study carried out by Steill. This makes the latter more generalizable, but not necessarily to all contexts, especially in other countries were the demographics of trauma patients could be different.

Both methods have the same value for true-positive and false-negative rates, such that 9 out of 10 people suffering cervical spine injuries were diagnosed by the evaluations, and only 1 patient did not need a radiography.

In this study, we observed higher true-negative rates in the assessment method using NEXUS guidelines. The rate of false positives obtained using NEXUS guidelines is lower than the rate obtained with CCR guidelines. Therefore, in the evaluation using the initial criteria, NEXUS guidelines perform better for evaluating patients (more specific).

The sensitivity for NEXUS and CCR guidelines are both 90%. However, in the survey conducted about specificity, NEXUS guidelines showed 54.73% specificity, while the specificity for CCR guidelines was 44.2%. In the study carried out by Zoe et al. (2012), it was shown that CCR guidelines are more sensitive than NEXUS guidelines for evaluation of patients. Therefore, the difference between the results of the two studies could be due to differences in sampling methods; the sampling method in Zoe’s study was retrospective, and 15 studies that examined these two guidelines were reviewed, while in this study, 200 patients were evaluated prospectively by the guidelines.

In the study of Stiell (2003), the sensitivity was 99.4% and 90.7%5 for CCR and NEXUS guidelines, respectively. They are similar to the values obtained in this study, which is an approval for the sensitivity range in these guidelines. Additionally, in a study conducted by Hoffman et al., a sensitivity of 99.6% was suggested for NEXUS guidelines, which is consistent with the results of our study and confirms the present findings [2,23].

The specificity for CPR guidelines in the study of Stiell [32] was reported to be 40.4%, which matches our study, but the specificity for NEXUS guidelines was 36.8%. The difference between the value obtained by Stiell and the value calculated in the present study could be caused by differences in the mechanism of damage in patients; in the study conducted by Steill et al., as the severity of the damage could act as a confounding factor in performing the guidelines on the patient [32], the most frequent cause of damage was motorcycle-car accidents, followed by simple rear vehicle accidents. However, in this study, the most frequent reason for referral was car accidents, pedestrian accidents, rollovers and falls from heights.

Goddard reported in a study that CCR has a clear superiority in reducing unnecessary radiographic imaging in conscious adults with stable status and cervical spine injury [28]. Ian et al. also demonstrated that in conscious patients who are stable, CCR criteria are preferred to NEXUS criteria [29]. Steill et al. also noted that CCR guidelines are superior for evaluating conscious patients.

The present study showed that the two guidelines have the same sensitivity for dealing with trauma patients and evaluating their need for radiography. It seems that the NEXUS guidelines have the same performance for handling trauma patients who need to undergo radiography. They also perform better than CCR guidelines in terms of eliminating cases that require no further radiologic investigations.

It must be kept in mind that written guidelines have never been non-negligible for the management of patients, and they can be changed according to the needs of the center and the study conditions. Guidelines’ efficiency can also be improved in some cases, and this is important in countries with limited resources and limited infrastructure for medical diagnostic interventions. To the best of our knowledge, this study was among the first comparing the two methods in patients living in a developing country. Utilizing NEXUS could cut costs for both unnecessary bioimaging and staff education. Additionally, using a single method for evaluating patients enables clinicians to communicate more effectively, especially in settings in which a unified health information management system is nonexistent [34–35]. In conclusion, our results show that using the NEXUS is at worst as sensitive and as specific as CCR, but at the same time easier to perform.

The limitations of the present study are that it was conducted in a single study center. Additionally, the center in question was a tertiary medical educational center, and it acted as the trauma referral center for areas northwest of Iran. This means that all of the evaluations, using either NEXUS or CCR guidelines, were performed by medical specialists or residents. In most circumstances, the first contact with a trauma patient is made by EMS personnel or general practitioners in less specialized centers, so the results should not be overgeneralized to first contact incidences. Another limitation of the study was the small sample size.

Finally, we recommend future studies to consider additional factors, such as the type of trauma, the time between the beginning of trauma and the patient’s referral, the assessing person, and the patients’ assessment convenience when evaluating which guidelines to use. Region-based effective factors, such as the number of cases referred, the workload, the number of participating medical staff, the individuals’ familiarity with the management of guidelines and the comfort level of the doctors must be evaluated.

## Supporting information

S1 File SPSS file of the study. the SPSS file depicting the data obtained during the study.
(SAV)
